# Sm16, A *Schistosoma mansoni* Immunomodulatory Protein, Fails to Elicit a Protective Immune Response and Does Not Have an Essential Role in Parasite Survival in the Definitive Host

**DOI:** 10.1155/2019/6793596

**Published:** 2019-12-01

**Authors:** Wilma Patrícia de Oliveira Santos Bernardes, Juliano Michel de Araújo, Gardênia Braz Carvalho, Clarice Carvalho Alves, Aline Thaynara de Moura Coelho, Isabela Thamara Sabino Dutra, Sueleny Silva Ferreira Teixeira, Rosy Iara Maciel de Azambuja Ribeiro, Marina de Moraes Mourão, Rosiane Aparecida da Silva-Pereira, Cristina Toscano Fonseca

**Affiliations:** ^1^Laboratório de Biologia e Imunologia de Doenças Infeciosas e Parasitárias, Instituto René Rachou, Fiocruz-MG, Belo Horizonte, Minas Gerais 30190009, Brazil; ^2^Laboratório de Helmintologia e Malacologia Médica, Instituto René Rachou, Fiocruz-MG, Belo Horizonte, Minas Gerais 30190009, Brazil; ^3^Laboratório de Patologia Experimental, Universidade Federal De São João Del Rei-Campus Divinópolis, Minas Gerais 35501-296, Brazil; ^4^Laboratório de Diagnóstico e Terapia de Doenças Infecciosas e Oncológicas, Instituto René Rachou, Fiocruz-MG, Belo Horizonte, Minas Gerais 30190009, Brazil

## Abstract

Sm16 is an immunomodulatory protein that seems to play a key role in the suppression of the cutaneous inflammatory response during *Schistosoma mansoni* penetration of the skin of definitive hosts. Therefore, Sm16 represents a potential target for protective immune responses induced by vaccination. In this work, we generated the recombinant protein rSm16 and produced polyclonal antibodies against this protein to evaluate its expression during different parasite life-cycle stages and its location on the surface of the parasite. In addition, we analyzed the immune responses elicited by immunization with rSm16 using two different vaccine formulations, as well as its ability to induce protection in Balb/c mice. In order to explore the biological function of Sm16 during the course of experimental infection, RNA interference was also employed. Our results demonstrated that Sm16 is expressed in cercaria and schistosomula and is located in the schistosomula surface. Despite humoral and cellular immune responses triggered by vaccination using rSm16 associated with either Freund's or alum adjuvants, immunized mice presented no reduction in either parasite burden or parasite egg laying. Knockdown of *Sm16* gene expression in schistosomula resulted in decreased parasite size *in vitro* but had no effect on parasite survival or egg production *in vivo*. Thus, our findings demonstrate that although the vaccine formulations used in this study succeeded in activating immune responses, these failed to promote parasite elimination. Finally, we have shown that Sm16 is not vital for parasite survival in the definitive host and hence may not represent a suitable target for vaccine development.

## 1. Introduction

Schistosomiasis is a neglected tropical disease with an important impact on public health [1]. The development of an effective vaccine formulation against the disease would help to control its transmission [2, 3]. However, this is hampered by the complexity of both the parasite and its life cycle [4, 5], as well as by the poor knowledge regarding the biological function of vaccine target antigens, and the mechanisms and components of the host's immune system involved in parasite elimination [6].

The skin is the first barrier confronted by schistosome parasites during infection of the definitive host. In fact, how parasites deal with immune factors in the skin dictates their survival in the host [7, 8]. Though the host's skin contains many cells that can respond upon parasite activation, schistosomes have evolved several mechanisms to evade host immune responses. The secretion of immunomodulatory molecules by the parasite's acetabular gland, resulting in increased IL-10, IL-1ra, and PGE production by host cells, is one of such mechanisms [9–11]. Therefore, immune modulatory proteins secreted by the parasite represent an interesting target for the hosts' protective immune response induced by immunization.

Sm16, also known as SPO-1 or SmSLP, is one of the most abundant components of *S. mansoni* cercarial excretion/secretion product [12]. This 16 kDa protein, which is secreted by the parasite during penetration of the mammalian host, shares 100% identity with its ortholog in *S. japonicum* [13]. Both orthologs are believed to play an important role in the suppression of cutaneous inflammatory responses during parasite penetration of the host skin [10, 13], thus facilitating parasite survival. Among the modulatory mechanisms induced by Sm16, inhibition of IL-2 production by lymph node cells from *S. mansoni* infected mice and increased production of IL-1ra by human keratinocytes have been described [10]. Additionally, Sm16 inhibits macrophage activation (due to retention of internalized antigen in early endosomes, causing a delay in antigen processing and presentation), consequently inhibiting the activation of the host adaptive immune response [14]. Sm16 also inhibits TLR-3 and TLR-4 signaling in human monocytic cell lines [15] and exerts an immunodulatory function even under LPS stimulation, inhibiting neutrophil infiltration to the site of LPS inoculation [16].

Although several studies have shown that Sm16 and its *S. japonicum* ortholog modulate inflammation *in vitro* and *in vivo* [10, 13–15, 17], the exact function of these proteins in the establishment of parasitism in the host is still not well understood. Herein, we expressed a recombinant form of Sm16 (rSm16) and raised polyclonal antibodies against it. We then evaluated the expression of Sm16 during the different parasite life-cycle stages associated with the definitive host and also evaluated the presence of this antigen on the parasite surface. The effect of the immune response triggered against Sm16 on parasite reproduction and survival was evaluated. Moreover, we explored the biological function of this protein during the course of experimental infection using RNA interference- (RNAi-) based gene knockdown. We observed that Sm16 is mainly expressed in the schistosomula life-cycle stage and is located on the external surface of the parasite. Although immunization of mice with different vaccine formulations was able to activate both cellular and humoral arms of the immune response, both formulations failed to induce protective immunity. Finally, knocking down the expression of Sm16 resulted in a decreased schistosomula size until day 4 of parasite culture *in vitro*, but the lack of Sm16 expression had no effect on parasite survival or egg production *in vivo*.

## 2. Methods

### 2.1. Mice and Parasites

Balb/c male and female mice (*Mus musculus*) aged 6-8 weeks were obtained from the Institute René Rachou (CPqRR)/FIOCRUZ (Fundação Oswaldo Cruz) animal facility. The *Schistosoma mansoni* LE strain is routinely maintained in the Mollusk Room “Lobato Paraense” at Instituto René Rachou (FIOCUZ/MG). *Schistosoma mansoni* cercariae were obtained by exposing infected *Biomphalaria glabrata* snails to light for 1-2 hours to induce shedding. For RNAi assays, and western blotting analysis, cercariae were mechanically transformed into schistosomula of cercariae [18] and were cultured in Glasgow Mem (GMEM) (Sigma-Aldrich, Germany) supplemented as previously described [19]. Infected mice were perfused and adult worms were recovered from the hepatic portal system, while the livers of the same animals were removed for egg recovery. Protocols using animals were licensed by the Ethics Committee of Animal Use (CEUA) of FIOCRUZ under licenses LW25/15 and LW22/16.

### 2.2. Recombinant Antigen Preparation

The fragment of the DNA sequence corresponding to the region encoding amino acids 23 to 90 of the *S. mansoni* Sm16 protein (GenBank: AAD26122.1 and WormBase ParaSite: Smp341790) was used to construct a synthetic gene for expression in *Escherichia coli*. According to Holmfeldt and colleagues [20], this sequence results in a recombinant protein, which is less prone to aggregation and is highly expressed in *E. coli*. A bacterial codon-optimized synthetic *Sm16* gene containing the restriction sites for the enzyme *BamH*I and *Xho*I at the 5′ and 3′ ends, respectively, was obtained from a commercial supplier (GenScript, Piscataway, NJ, USA) inserted into a pUC57 vector. An initiation codon ATG was inserted between the *BamH*I restriction site and the codon corresponding to the first amino acid of rSm16. This construct was subcloned into the *BamH*I/*Xho*I sites of the pET21a plasmid (Novagen) and transfected into *E. coli* BL21 (DE3).

In order to express and obtain rSm16, transformed cells were cultured overnight at 37°C in liquid LB medium (Kasvi) supplemented with 100 *μ*g/ml ampicillin (Sigma-Aldrich). A volume of 10 ml of this overnight starter culture was used to inoculate 1000 ml of fresh LB medium containing 100 *μ*g/ml ampicillin. When the OD_600_ reached approximately 0.6, protein expression was induced by the addition of 1 mM IPTG (Promega) and allowed to proceed for 4 hours at 37°C. Cells were harvested by centrifugation at 5000 × g for 20 minutes and the supernatant was discarded. The cell pellet was resuspended in lysis buffer (50 mM Tris (GE-Healthcare), 500 mM NaCl (Êxodo Científica), 0.2 mM EDTA (Química Moderna), 3% sucrose (Synth), and 1% TritonX-100 (Sigma-Aldrich)), containing 200 *μ*g/ml lysozyme (Sigma-Aldrich), 1 mM phenylmethylsulfonyl fluoride (PMSF) (GE Healthcare), and 20 *μ*g/ml deoxyribonuclease I from bovine pancreas type IV (DNase) (Sigma-Aldrich). Proteins were extracted by sonication in an ultrasonic processor (VC 750 Vibra-Cell™) by 5 cycles of a 30 sec pulse using 30% output followed by 1 min on ice. The lysate was clarified by centrifugation at 15000 × g for 30 minutes and the supernatant collected for His-tag protein purification by affinity chromatography using a nickel column QIAexpress Ni-NTA Fast Start Kit (Qiagen) under denaturing conditions. The purified protein was dialyzed against PBS at pH 7.2 using the Mini Dialysis Kit 1 kDa cut-off (GE HealthCare) and quantified using the BCA Protein Assay Kit (Thermo Scientific Pierce, Rockford, IL, USA).

The expression and purification of rSm16 were analyzed by 15% SDS-PAGE, as described by Laemmli [21]. The protein was blotted onto nitrocellulose membrane (GE Healthcare) as described by Towbin et al. [22], followed by blocking with 5% dry milk at 4°C for 16 hr. After washing, the membrane was incubated with monoclonal 6x-His-tag antibody (1 : 3,000) (GE Healthcare) for 1 hr at room temperature, and after three washes in 0.05% Tween 20 in TBS (TBS-T) (LCG Biotecnologia), the membrane was incubated for 1 hr with horseradish peroxidase- (HRP-) conjugated goat anti-mouse IgG antibody (SouthernBiotech) (1 : 5,000). The reaction was developed using 3-3′-diaminobenzidine tetrahydrochloride substrate (Sigma-Aldrich).

### 2.3. Production of Anti-Sm16 Polyclonal Antibodies

In order to obtain Sm16-specific polyclonal antibodies, female Balb/c mice (six to eight weeks) were inoculated subcutaneously with 25 *μ*g of rSm16 plus Freund's complete adjuvant in the first dose and Freund's incomplete adjuvant in the two subsequent doses (with a 15-day interval between each dose). The control group was inoculated with saline plus Freund's adjuvant. Serum samples were obtained every 15 days after the first immunization and titrated by enzyme-linked immunosorbent assay (ELISA).

### 2.4. Western Blotting Analysis

Protein extracts from different stages of the *S. mansoni* life cycle, cercariae, 3-hour cultured schistosomula, 7-day cultured schistosomula, adult worms, and eggs, were obtained by lysis of the parasites in lysis buffer (8 M urea, 2 M thiourea, 4% CHAPS, 20 mM Tris, 500 mM DTT, and protease inhibitor (GE Healthcare)). After homogenization under continuous agitation for 2 hrs at room temperature, followed by 10 repeated passages through a 31G hypodermic needle, the homogenate obtained was centrifuged at 20,000 × g for 30 min at 25°C and the supernatant was collected. Protein extraction from eggs was improved by crushing eggs with a pestle. After protein quantification using the Bradford protein assay, 10 *μ*g of protein extract from each stage was loaded into two identical 15% SDS-PAGE gels. One gel was stained by Coomassie Blue R-250, and the other gel was blotted onto the nitrocellulose membrane, as described above. The membrane was blocked for 16 hours at 4°C and incubated with hyperimmune serum against rSm16 (1 : 200) for 1 hr. After the washing step, the membrane was incubated for 1 hr with a horseradish peroxidase- (HRP-) conjugated goat anti-mouse IgG antibody (Amersham ECL Anti-Mouse IgG) diluted 1 : 5,000. After a further washing step, protein expression was detected with a chemiluminescent substrate using the ECL Prime Western Blotting Detection Reagent (GE Healthcare) using an ImageQuant LAS 4000 (GE Healthcare). The quantification of protein expression was performed by densitometric analysis using the ImageJ software (version 1.51p), and the Coomassie Blue-stained SDS-PAGE gel was used to normalize protein loading.

### 2.5. Immunolocalization of Sm16

Schistosomula were incubated for either 90 min or 3 hrs in supplemented RPMI medium (3% streptomycin/penicillin and 5% FBS) or seven days in GMEM supplemented medium. Approximately 1,000 schistosomula were fixed with 1% formaldehyde in PBS for 1 hr at 4°C. The schistosomula were then incubated in blocking solution (1% BSA in PBS) for 30 min and then incubated with agitation with either anti-Sm16 hyperimmune or control serum diluted 1 : 100 in blocking solution for approximately 16 hrs. After a washing step, the schistosomula were incubated for 2 hrs at room temperature with PE-conjugated rat anti-mouse IgG1 antibody (BD Pharmingen) diluted 1 : 80 in blocking solution. As a specificity control, some schistosomula were incubated with only the secondary antibody. The parasites were washed with PBS, mounted in antifading medium, and examined using a fluorescence microscope (LSM 510 Carl Zeiss).

### 2.6. Vaccination Protocol

Female six-eight-week-old Balb/c mice were immunized by subcutaneous route (12 animals/group) with rSm16 (25 *μ*g/animal/dose) plus alum adjuvant (1 mg/animal/dose), or Freund's adjuvant (100 *μ*l/animal/dose). In the first dose, mice were immunized with complete Freund's adjuvant (CFA), and in the subsequent boosters, incomplete Freund's adjuvant (IFA) was used. The control groups were inoculated with saline plus alum or saline plus Freund's adjuvant. Mice received three doses in a fifteen-day interval regimen. Animals were challenged through percutaneous infection with 100 cercariae (LE Strain) 15 days after the last dose. Fifty days after infection, animals were perfused by portal veins and adult worms were obtained as previously reported [23, 24] ([Supplementary-material supplementary-material-1]). Briefly, animals were euthanized; a solution containing saline plus 500 units/l of heparin was pumped into the aortic artery and worms were collected from the hepatic portal vein. The number of worms was counted using a stereomicroscope. The protection levels were calculated by comparing the number of worms recovered from the immunized group with its respective control group using the following formula:

(1) $$\begin{array}{c} \text{PL}=\frac{\text{BCG}-\text{BIG}}{\text{BCG}}\times 100, \end{array}$$

where PL is the protection level, BCG is the parasite burden of the control group, and BIG is the parasite burden of the immunized group. The results were analyzed using unpaired Student's *t*-test with 95% confidence level.

### 2.7. Histopathological Analysis and Egg Counts from the Gut and Liver

The gut and liver from each mouse from both the control and rSm16-immunized groups were removed after perfusion. These organs were weighed and digested with 10% KOH for 16 hrs at 4°C and for 30 min at 37°C. The eggs were obtained by centrifugation at 900 × g for 10 min and resuspended in 1 ml of saline. The number of eggs was counted using a light microscope. The results were analyzed using either the unpaired Student's *t*-test or the Mann-Whitney test for parametric and nonparametric data. For both tests, a 95% confidence level was used.

In order to evaluate the effect of immunization on granuloma formation, a section of the left lateral lobe of the liver of control and rSm16-immunized mice were collected and fixed in 4% buffered formaldehyde in PBS. Histological sections were performed using a microtome and the slides were stained with Gomory trichrome. The granuloma area was determined as described by Alves and collaborators [25]. Briefly, approximately 100 granulomas were evaluated for each group. Only granulomas at the exudative-productive stage with a well-defined egg were evaluated using a 10x objective lens. The granuloma area was calculated using the AxioVision version 4.8 image analysis software (Carl Zeiss MicroImaging GmbH, Germany) and expressed in square micrometers (*μ*m^2^). The results were analyzed using either the unpaired Student's *t*-test or the Mann-Whitney test for parametric and nonparametric data. For both tests, a 95% confidence level was used.

### 2.8. Antibody Assessment

Individual sera from the mice in each immunized group were obtained 15 days after each immunization dose. ELISA was performed to evaluate the production of specific anti-rSm16 IgG, IgG1, and IgG2a antibodies. Briefly, MaxiSorp 96-well microtiter plates (Nunc, USA) were coated with 5 *μ*g/ml rSm16 in carbonate-bicarbonate buffer (pH 9.6) for 16 hrs at 4°C. The plates were blocked with 300 *μ*l/well of 0.05% Tween-20 in phosphate-buffered saline (pH 7.2) (PBST) plus 10% FBS for 2 hrs at room temperature. One hundred microliters of each serum sample, diluted 1 : 800 in PBST to evaluate IgG and IgG1, or 1 : 400 for IgG2a, were added to the plates and incubated for 1 hr at room temperature (RT). After six washes with PBST, HRP-conjugated goat anti-mouse IgG (1 : 10,000), IgG1, (1 : 10,000), and IgG2a (1 : 12,000) (Southern Biotech, USA) were added and incubated for 1 hr at RT. The color reaction was obtained by addition of TMB substrate (Microwell Peroxidase Substrate System, Bio-Rad, USA) and stopped with 5% sulfuric acid. The absorbance was detected at 450 nm using an ELISA plate reader (Bio-Rad, USA), and the data were analyzed using a two-way ANOVA (*P* < 0.05) followed by Tukey's multiple comparison test with a 95% confidence level. Endpoint antibody titers were determined using a pool of sera samples from each group. The pool of sera from each group was serially diluted in PBST from 1 : 50 to 1 : 1,638,400. The cut-off point for seropositivity was determined using the mean absorbance observed in blank wells plus two standard deviations. The endpoint titer was determined as the last dilution in which the observed absorbance was above the cut-off point.

### 2.9. Immunophenotypic Analysis

Analysis of the cellular immune response was performed using blood samples from mice, collected 15 days after the third vaccine dose. Red cells were lysed using ACK lysing buffer, washed twice with apyrogenic saline, and adjusted to 1 × 10^6^ cells/well. Thereafter, cells were incubated with anti-CD16/CD32 antibodies (clone 2.4G2, BD Bioscience, USA) to block antibody binding to Fc*γ*R. Surface molecules were labeled by incubating the cells for 30 min with monoclonal antibodies using one of the following two combinations: (i) anti-CD19 conjugated to PE-Cy7 (clone IM7, BD Pharmingen), anti-CD3 conjugated to FITC (clone BM8, eBioscience), and anti-CD27 conjugated to biotin (clone LG.7F9, eBioscience) or (ii) anti-CD4 conjugated to FITC (clone GK1.5, BD Pharmingen) and anti-CD44 conjugated to APC (clone IM7, BD Pharmingen). Then, the cells were washed, and those of the first combination were incubated for 20 min with streptavidin APC-Cy7 (1 : 1000) for 15 min at 4°C. After a washing step, cells were acquired using a LSRFortessa flow cytometer (Becton Dickinson, San Jose, CA). Data were analyzed using FlowJo software 10.0 (Tree Star, Ashland). The results were analyzed using one-way ANOVA or Kruskal-Wallis test followed by Holm-Sidak's or Dunn's multiple comparison tests for parametric and nonparametric data, respectively. For both tests, a 95% confidence level was used. Samples that had a reduced number of acquired events, interruption of the flow during acquisition, or an excessive number of doublets were excluded from the analysis.

### 2.10. Cytokine Analysis

To assess cytokine production, plasma obtained 15 days after the last immunization dose was used in CBA-based flow cytometry. For this, the anti-mouse Cytometric Bead Array (CBA) Th1/Th2/Th17 Kit (BD Pharmingen, USA) was used according to the manufacturer's protocol. Data acquisition was performed in a FACSVerse flow cytometer (BD, USA) and analyzed using FCAP Array Software (Becton Dickinson). The results were analyzed using either one-way ANOVA or the Kruskal-Wallis test, followed by either Holm-Sidak's or Dunn's multiple comparison tests, for parametric and nonparametric data, respectively. For both tests, a 95% confidence level was used.

### 2.11. Double-Stranded RNA Synthesis and Parasite Exposure

The sequences of the primers used to amplify the fragments for double-stranded RNA (dsRNA) synthesis and quantitative real-time PCR (RT-qPCR) were designed using the Primer 3 program (v.0.4.0) (bioinfo.ut.ee/primer3-0.4.0) and purchased from Integrated DNA Technologies Inc. (Coralville, IA). A T7 promoter sequence was added to the 5′-end of the dsRNA's designed primers. A 248 bp fragment of *Sm16* gene was amplified by PCR using the following primers: forward 5′aatacgactcactatagggCCTCACCCGAGTGAAAAAGA3′ and reverse 5′taatacgactcactatagggAATCCTTGGAAGACGCATTG3′. Total RNA from schistosomula was used for cDNA synthesis, and the latter as a template for *Sm16* amplification. Thereafter, the amplicon was Sanger sequenced to confirm the *Sm16* fragment identity using the BigDye™ Terminator v3.1 Cycle Sequencing Kit and an ABI 3730 DNA Analyzer. As a RNAi nonspecific control, a 360 bp fragment of the green-fluorescent protein (GFP) gene was amplified from the pCRII-GFP plasmid vector (Thermo Fisher Scientific, USA) using the primers GFP_dsRNA_Forward 5′taatacgactcactatagggGTGTTCAATGCTTTGCGAGA3′ and GFP_dsRNA_Reverse 5′taatacgactcactatagggCTTTTCGTTGGGATCTTTCG3′.

The dsRNAs were synthesized using the PCR products and the T7 RiboMAX™ Express RNAi System Kit (Promega, USA), according to the manufacturer instructions. The syntheses were performed overnight at 37°C. Sample concentration was determined using a Nanodrop Spectrophotometer ND-1000 (Thermo Fischer Scientific, USA), and the integrity of the dsRNA was assessed by 1% agarose gel electrophoresis.

Mechanically transformed schistosomula were exposed to 200 nM of dsRNA (*Sm16* or *GFP*) in 6-well plates containing approximately 12,000 parasites in supplemented GMEM medium (3,000/ml). The same number of parasites was incubated in supplemented GMEM only. The cultures were maintained at 37°C, 5% CO_2_, and 95% humidity for up to seven days after dsRNA exposure.

### 2.12. Gene Expression Analyses

Each day, 4,000 schistosomula were removed from the cultures used for RNAi for the analysis of relative gene expression using quantitative real-time PCR (RT-qPCR). The RNA extractions were carried out using TRIzol Reagent (Thermo Fisher Scientific, USA), as recommended by the manufacturer. Residual DNA was removed by DNase digestion using Turbo DNase (2 U/*μ*l) (Thermo Fisher Scientific, USA). RNA was quantified using the Qubit 2.0 Fluorometer (Thermo Fischer Scientific, USA), and cDNA was synthesized using the ImProm-II™ Reverse Transcription System (Promega), according to manufacturer instructions. The RT-qPCR experiments were carried out in technical triplicates in the ViiA7 System (Thermo Fischer Scientific, USA) using Power SYBR® Green Master mix (Applied Biosystems, USA) and primers to amplify a 72 pb *Sm16* fragment (forward 5′ACGCCAATTATCTTCGCTGT3′ and reverse 5′TTGCTCTAACGTTGTAGCTGTGA3′). A fragment of 53 pb from *Smgapdh* (Smp-056970) was used as endogenous normalization control (forward 5′TCGTTGAGTCTACTGGAGTCTTTACG3′ and reverse 5′AATATGAGCCTGAGCTTTATCAATGG3′). The reactions were performed in a 20 *μ*l final volume and after performing a concentration curve following the MIQE guideline recommendations [26]. The optimal concentration of primers was established 1500 nM for the *Sm16* and 900 nM for the *gapdh*. Post-RNAi *Sm16* transcript levels were assessed using the relative 2^-*ΔΔ*Ct^ method [27] and calculated as a percentage of difference compared to the *GFP* unspecific control. Experiments were conducted at a Real-Time PCR Facility/RPT09D PDTIS/René Rachou Institute/FIOCRUZ MG.

### 2.13. In Vitro and In Vivo RNAi Experiments

Schistosomula exposed to medium only, or *Sm16* or *GFP* dsRNA, were examined every two days for evaluation of phenotypic alterations using an inverted fluorescence microscope (Axio Observer, Carl Zeiss). Characteristics, such as parasite surface area, color, motility, and viability, were evaluated. Images of two fields from each sample, containing approximately 100 schistosomula, were captured, and the area (*μ*m^2^) of each schistosomulum was determined using AxioVision version 4.8 software. Four independent experiments were performed to generate the data that was analyzed using a Kruskal-Wallis test followed by Dunn's multiple comparison test at a 95% confidence level.

To monitor parasite viability, 5 *μ*g/ml propidium iodide (Sigma-Aldrich) was added to approximately 100 schistosomula, and the stained parasites were observed using a fluorescence microscope with a 544 nm filter (Carl Zeiss). The results from four biological replicates were analyzed using two-way ANOVA followed by Tukey's multiple comparison test at a 95% confidence level.

After parasite exposure to *Sm16* or *GFP*-dsRNA for 2 days, approximately 270 schistosomula were subcutaneously inoculated into 6-to-8-week-old Balb/c male mice. Untreated schistosomula were also inoculated as a control. Overall, 12 mice were used for each of the 3 treatment groups, per each of the two independent biological replicates performed. After 50 days, adult worms were recovered by perfusion of the hepatic portal system from mice. The liver and gut from mice were also obtained, weighed, and digested with 10% KOH solution for subsequent determination of the egg number. The data were analyzed using either one-way ANOVA or the Kruskal-Wallis test, followed by either Holm-Sidak's or Dunn's multiple comparison tests, for parametric and nonparametric data, respectively. For both tests, a 95% confidence level was used.

## 3. Results

### 3.1. Expression and Purification of the rSm16 Protein

**Figure 1:**
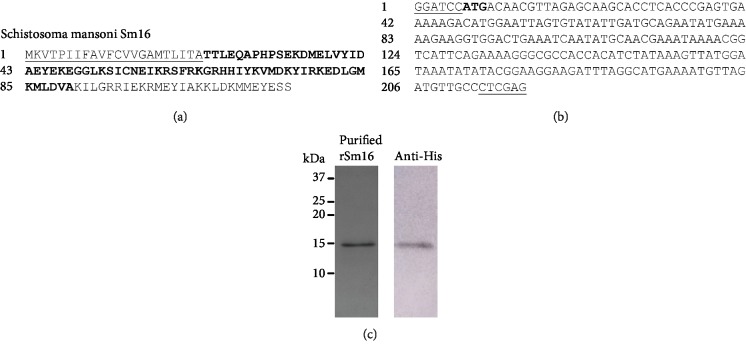
Purification of recombinant Sm16. (a) Amino acid sequence of *Schistosoma mansoni* Sm16 (GenBank: AAD26122.1 and WormBase ParaSite: Smp_341790). The signal peptide is underlined and the sequence in bold corresponds to amino acids 23 to 90. (b) Synthetic gene construction containing the DNA sequence corresponding to the region encoding the amino acids 23 to 90 with restriction enzyme sites *BamH*I and *Xho*I at the 5′ and 3′ ends, respectively, and the initiation codon ATG in bold. (c) 15% SDS-PAGE of purified rSm16 stained by Coomassie Blue R-250 and Western blot using monoclonal 6x-His-tag antibody. Molecular weight markers Dual Color (Bio-Rad) are indicated in kDa.

In order to obtain rSm16, we choose to express the fragment of the Sm16 protein corresponding to amino acids 23-90 in an *E. coli* system (Figure 1(a)). The DNA sequence of the synthetic gene was optimized for expression in prokaryotic systems through changing the native codons to those preferentially used by *E. coli*, as represented in Figure 1(b). A protein of approximately 15 kDa, corresponding to expected molecular weight of rSm16, was purified from the bacterial lysate after 4 hours of IPTG induction, using nickel-affinity purification. A high purity fraction of rSm16 was observed in a Coomassie Blue-stained SDS-PAGE (Figure 1(c)). The His-tag fused to rSm16 was recognized by a monoclonal 6x-His-tag antibody, indicating the correct expression of 15 kDa recombinant protein from the designed gene sequence (Figure 1(c)).

### 3.2. Sm16 Is Present in the Schistosomula Tegument and Is More Expressed in Cercariae and Early Transformed Larval Stages

Sm16 has previously been shown to have low immunogenicity in mice immunized with either the *Sm16* gene or recombinant protein [17]. Here, to test the immunogenicity of rSm16 (23-90), mice were immunized with three doses of recombinant antigen combined with Freund's adjuvant. Significant production of specific antibodies was observed in immunized mice after both the second and the third immunizations (SFig. [Supplementary-material supplementary-material-1]). The endpoint antibody titer observed after two immunization doses was 1 : 51,200, and 30 days after the third immunization, there was an increase of eightfold in the antibody's endpoint titer against rSm16.

**Figure 2:**
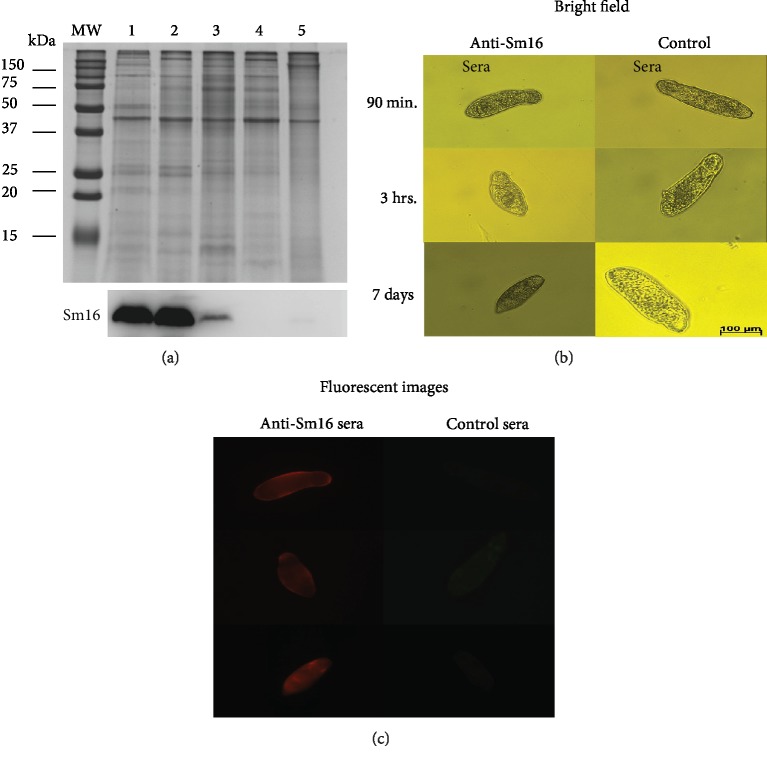
Expression and location of native *Schistosoma mansoni* Sm16. (a) 10 *μ*g of total protein extract from *S. mansoni* cercariae (1), 3-hour schistosomula (2), 7-day schistosomula (3), adult worms (4), and eggs (5) were electrophoresed in a 15% SDS-PAGE and stained by Coomassie Brilliant Blue R-250 or blotted onto nitrocellulose membranes for Western blot analysis using serum from mice immunized with the recombinant Sm16 protein. Densitometric analysis was performed using the ImageJ software, and the Coomassie Blue-stained SDS-PAGE gel was used to normalize protein loading. (b) Transformed schistosomula were maintained in culture for 90 min, 3 hrs, or 7 days and were incubated with control serum, from mice inoculated with saline+CFA/IFA, or with serum from mice immunized with rSm16+CFA/IFA. Antibody reactivity to Sm16 was detected by a PE-conjugated rat anti-mouse IgG1 antibody. The schistosomula were observed by fluorescence microscopy using a LSM 510 Carl Zeiss microscope.

The polyclonal antibodies obtained after the third immunization dose were used to assess the Sm16 expression throughout the parasite stages associated with the vertebrate host (cercaria, schistosomulum, adult worms, and eggs). Densitometric analysis demonstrated that cercariae and recently transformed schistosomula (3 hours) express approximately 2-fold higher levels of Sm16 than 7-day-old schistosomula. In adult worms and egg extract, no protein expression of Sm16 could be detected (Figure 2(a)). By immunofluorescence of whole fixed schistosomula, we detected Sm16 on the parasite surface. Antibodies against rSm16 specifically recognized the native form of this protein on the surface of schistosomula at 90 min, 3 h, and 7 days after transformation (Figure 2(b)). Neither the control serum (Figure 2(b)), nor the secondary antibody recognized Sm16 (data not shown).

### 3.3. rSm16/Alum Vaccine Formulation Induced a Stronger Humoral Immune Response than rSm16/Freund's

**Figure 3:**
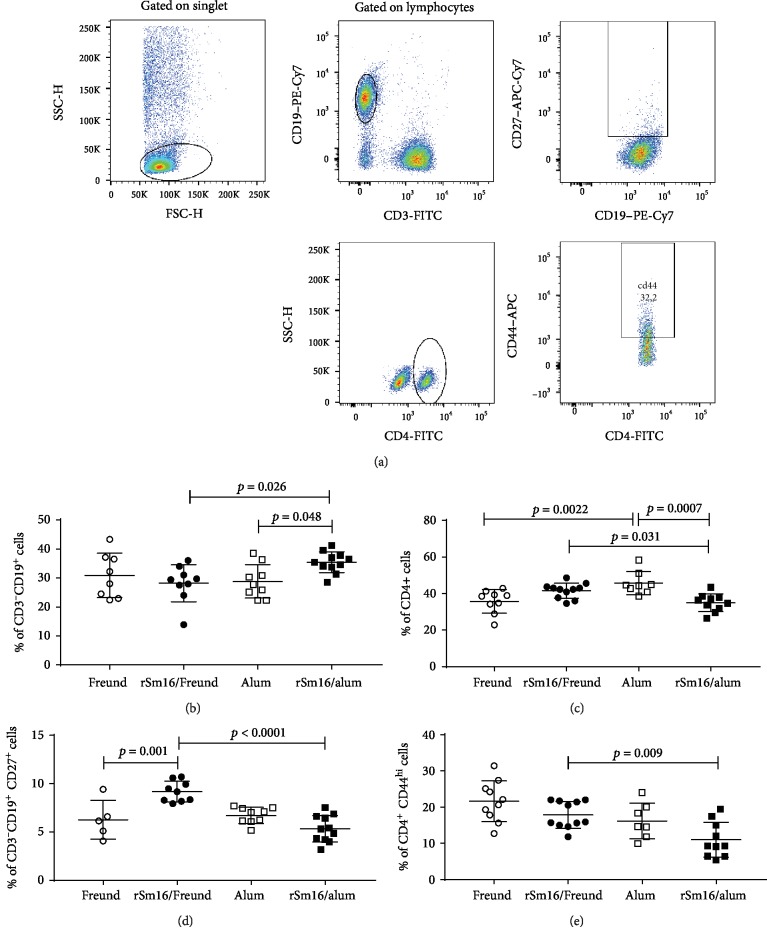
Immunophenotyping of blood cells from rSm16-immunized mice. Blood samples were obtained from 12 mice for each group to determine the frequency of total and memory B cells and total and effector T CD4^+^ cells. Data analysis was carried out as demonstrated in (a). Within the singlet cells/lymphocyte population, CD3^−^CD19^+^ B cells were selected and the percentage of total B cells was evaluated. Within the population of CD3^−^CD19^+^ double-positive cells, the percentage of CD19^+^CD27^+^ (memory B cells) was determined. Within the singlet cell/lymphocyte population, total T CD4^+^ were assessed and the frequencies were defined. Within that population, the percentage CD4^+^CD44^+^ representing CD4^+^ T effector cells was determined. Data represents percentage of CD3^−^CD19^+^ B Cells (b), CD4^+^ T cells (c), CD19^+^CD27^+^ memory B cells (d), and CD4^+^CD44^+^ effector T cells (e) in mice immunized with Freund's adjuvant only (closed circles), rSm16/Freund's (black circles), alum only (open squares), and rSm16/alum (black squares). Mean ± SD is presented in each graph. Significant differences which were observed using one-way ANOVA (c–e) or Kruskal-Wallis tests (b) followed by Holm-Sidak's (c–e) or Dunn's (b) multiple comparison tests, using a correction for multiple comparison analysis, are shown in the figures.

The ability of the Sm16 recombinant protein to elicit an immune response in the vertebrate host was evaluated using two distinct vaccine formulations: rSm16 administered with either Freund's adjuvant or alum. Immunophenotyping was performed using the blood from mice 15 days after their third immunization. The strategy used to analyze the data is presented in Figure 3(a). The rSm16/alum formulation induced a higher proportion of circulating B cells (CD3^−^CD19^+^) and a lower proportion of circulating T CD4^+^ cells (CD3^+^CD4^+^) (Figures 3(b) and 3(c)) than either alum alone or Sm16/Freund's formulations. Mice immunized with Sm16/Freund's formulation showed a higher proportion of memory B cells compared to either Freund's alone or rSm16/alum formulations (Figure 3(d)). A higher proportion of CD4^+^ effector cells was also observed with the Sm16/Freund's formulation compared to Sm16/alum (Figure 3(e)).

**Figure 4:**
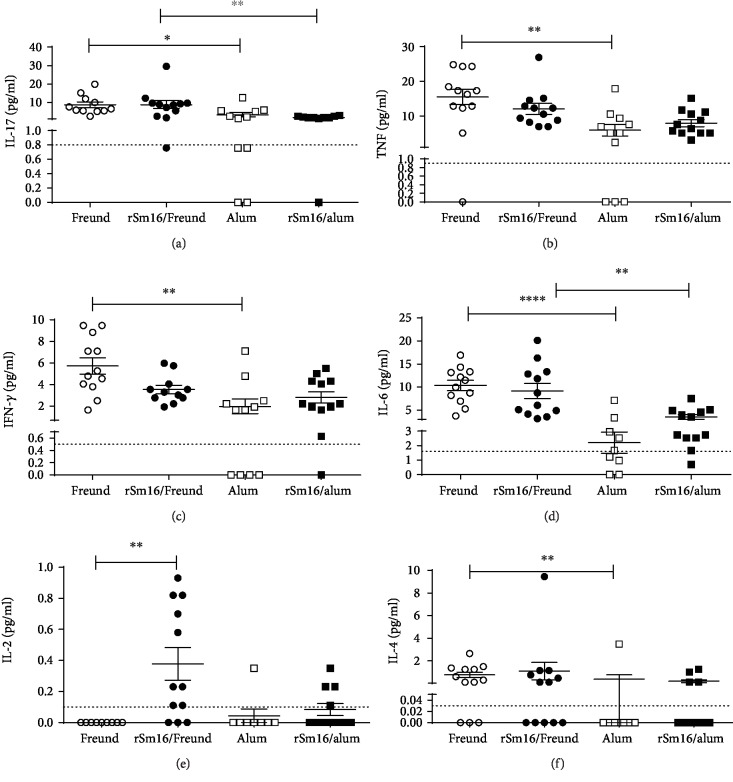
Cytokine profile induced in Balb/c mice immunized with rSm16. Plasma from mice immunized with different vaccine formulations was obtained fifteen days after the third immunization for cytokine measurement. (a) IL-17, (b) TNF-*α*, (c) IFN-*ɣ*, (d) IL-6, (e) IL-2, and (f) IL-4 production in response to immunization with Freund's adjuvant only (open circles), alum only (open squares), rSm16/Freund's (black circles), or rSm16/alum (black squares). Cytokine levels were measured using the CBA Th1/Th2/Th17 kit. Mean ± SEM is presented in the graph. Significant differences observed using one-way ANOVA (d) or Kruskal-Wallis test (a–c,e, f) followed by Holm-Sidak's (d) or Dunn's (a–c,e, f) multiple comparison tests, using a correction for multiple comparison analysis, are shown in the figures. Statistically significant differences are denoted in each graph.

The levels of circulating cytokines in the plasma of mice inoculated with the different vaccine formulations or their respective controls were also determined. No differences in cytokine production were observed between the plasma from animals immunized with rSm16/alum in comparison with the alum-only control group (Figure 4). In contrast, mouse immunization with rSm16 plus Freund's adjuvant produced an increased level of IL-2 compared to animals inoculated with saline plus Freund's adjuvant (Figure 4(e)). In the absence of the recombinant antigen, alum and Freund's adjuvants induced different patterns of circulating cytokines. Freund's adjuvant induced higher levels of circulating IL-17, TNF-*α*, IFN- ɣ, IL-6, and IL-4 than alum (Figures 4(a)–4(f), respectively). Decreased levels of IL-17 and IL-6 cytokines were also observed in the plasma from mice that were immunized with Sm16/alum formulation compared to the levels of these cytokines observed in sera from Sm16/Freund's immunized mice (Figures 4(a) and 4(d), respectively). IL-10 levels were below the CBA detection limit and, therefore, could not be determined.

**Figure 5:**
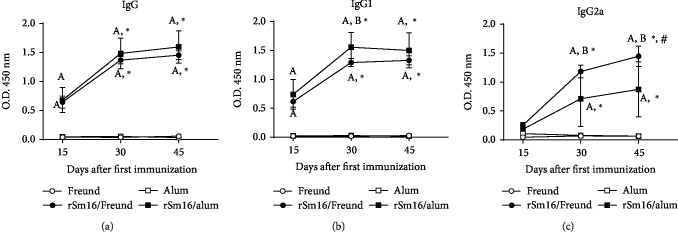
Production of Sm16-specific antibodies in immunized mice. Sera from mice were obtained 15 days after each immunization dose and were assessed to determine the levels of IgG (a), IgG1 (b), and IgG2a (c) antibodies against rSm16 in mice inoculated with Freund's adjuvant only (open circles), alum only (open squares), rSm16/Freund's (black circles), or rSm16/alum (black squares). Mean ± SD is presented in the graphs. Two-way ANOVA followed by Tukey's multiple comparison test was performed using a correction for multiple comparison analysis. Significant differences related to the first dose are indicated by an asterisk (*P* < 0.05) and to the second dose are denoted by symbol # (*P* < 0.05). Significant differences between the Freund's only and the alum-only control groups are denoted by the letter “A” (*P* < 0.05). Significant differences between rSm16/Freund and rSm16/alum are indicated by the letter “B” (*P* < 0.05).

Mouse immunization with rSm16 administered with either Freund's or alum adjuvant induced a significant production of specific anti-rSm16 IgG, IgG1, and IgG2a antibodies compared with their respective controls, as early as fifteen days after the first immunization dose for IgG and IgG1, and fifteen days after the second dose for IgG2a (Figure 5(a)–5(c), respectively). The levels of these antibodies, after the second and third immunization doses, increased with both formulations, in comparison to the levels observed fifteen days after the first immunization dose (Figure 5(a)–5(c)). After the second immunization dose, higher levels of anti-rSm16 IgG1 antibodies were observed in the rSm16/alum group compared to rSm16/Freund's group (Figure 5(b)). In contrast, mice immunized with rSm16/Freund's produced significantly higher levels of specific IgG2a than rSm16/alum immunized mice, after the second and third doses (Figure 5(c)). Also, after the first, second, and third immunization doses, the endpoint titer of anti-rSm16 IgG was 4-, 2-, and 2-folds higher, respectively, in mice that received the rSm16/alum formulation than those immunized with rSm16/Freund's (Table 1). Differences were also observed in the endpoint titer of rSm16-specific IgG1. Mice immunized with rSm16/alum produced 8-, 4-, and 2-folds more antibodies than the rSm16/Freund's group after the first, second, and third doses, respectively (Table 1). Both vaccine formulations produced similar titers of IgG2a against rSm16 throughout the immunization scheme.

### 3.4. Regardless of the Immune Response Elicited, Both rSm16 Vaccine Formulations Failed to Induce Protection against Challenge Infection

**Figure 6:**
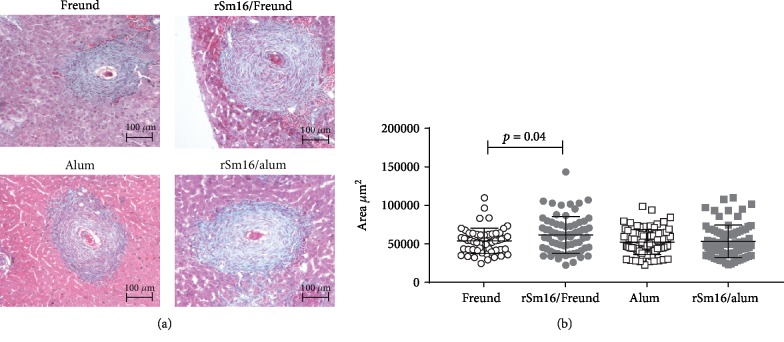
Hepatic granuloma area in mice immunized with rSm16. (a) Representative histological sections of liver granulomas from each immunized group. Scale bar = 100 *μ*m (100x). (b) Approximately 100 granulomas from rSm16/Freund's (gray circles) and its Freund's only control group (open circles) and from rSm16/alum (gray squares) and its alum-only control group (open squares) with a single well-defined egg at the exudative-productive stage were randomly selected and measured. Total area of the granulomas was expressed in square micrometers (*μ*m^2^). Mean ± SD is presented in the graphs. Unpaired Student's *t*-test was used to analyze data from Freund's only and rSm16/Freund's groups, and the Mann-Whitney test was used to analyze data from alum-only and rSm16/alum groups. Statistically significant difference is denoted in the graph.

To evaluate the impact of the immune response elicited by immunization of mice with rSm16 on the parasite survival, after receiving three doses of the vaccine, mice were challenged with *S. mansoni* cercariae, and fifty days postchallenge, the parasite burden was determined. Mouse immunization with rSm16 formulated with either Freund's or alum did not result in significant reduction in parasite burden or in the number of eggs per gram of liver or gut, in both immunization trials (Table 2). Liver pathology was also not affected by immunization with the rSm16/alum formulation. However, when the rSm16/Freund's formulation was used, an increase in granuloma size was observed in immunized mice compared to the control group (Figure 6).

### 3.5. Knockdown of *Sm16* in Schistosomula Did Not Impair Parasite Survival in the Vertebrate Host

**Figure 7:**
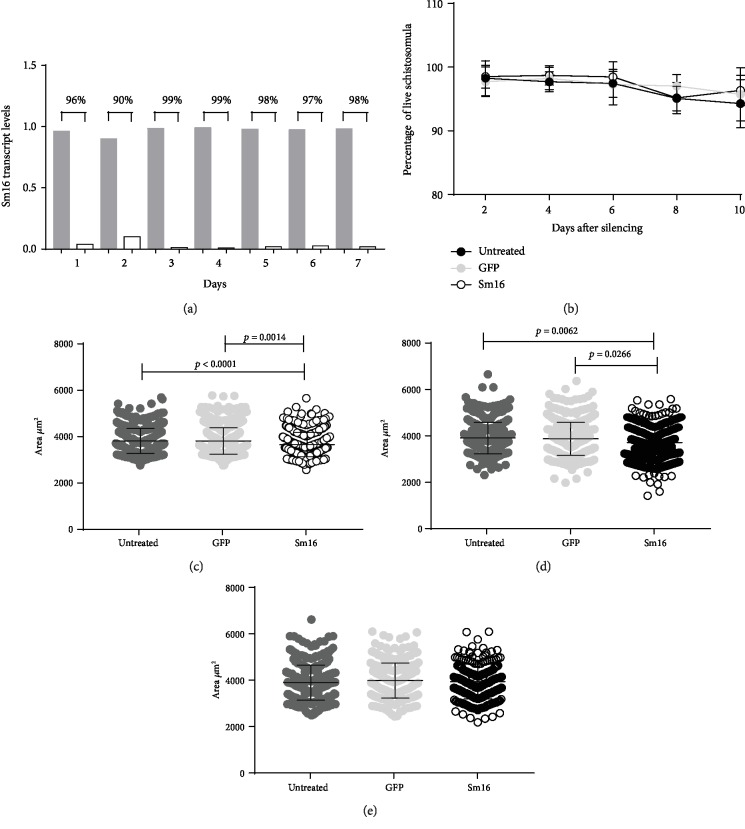
*In vitro* analyses of parasite development following *Sm16* knockdown. (a) Schistosomula were exposed to specific *Sm16* dsRNA and unrelated *GFP* dsRNA for seven days. The level of the *Sm16* transcript was assessed daily by RT-qPCR. Gray bars represent the expression of the Sm16 transcript on schistosomula exposed to *GFP* dsRNA and white bars to *Sm16* dsRNA. The percentage numbers represent the reduction of the *Sm16* transcript levels in schistosomula exposed to *Sm16* dsRNA relative to those exposed to *GFP* dsRNA. (b) Parasite survival after *Sm16* knockdown was evaluated *in vitro* every 2 days for 10 days. Approximately 100 schistosomula were labeled with 5 *μ*g/*μ*l propidium iodide and observed by fluorescence microscopy. The area of the schistosomula was evaluated two (c), four (d), and six (e) days after treatment with the dsRNA. Images of approximately 100 schistosomula were captured, and the area of each schistosomulum (*μ*m^2^) was measured using AxioVision version 4.8 software. Untreated parasites are denoted by dark gray circles. Light gray circles correspond to the control *GFP* dsRNA-treated schistosomula and open circles to specific *Sm16* dsRNA-treated schistosomula. Mean ± SD is presented in the graph. The data were generated from four independent experiments and analyzed using a two-way ANOVA followed by Tukey's multiple comparison test (b) or Kruskal-Wallis test followed by Dunn's multiple comparison test (c–e) using a correction for multiple comparison analysis. Statistically significant differences are shown in the figures.

The use of rSm16 in vaccine formulations had no effect on parasite elimination, although an immune response was elicited by vaccination. Therefore, to further evaluate the role of Sm16 in parasite biology, we knocked down *Sm16* expression in schistosomula using RNAi methodology. Using specific *Sm16* dsRNA, the levels of *Sm16* transcripts were significantly reduced (96%) as early as 24 hours after schistosomula treatment (Figure 7(a)). The *Sm16* transcript level remained reduced (98%) until the 7^th^ day post-dsRNA treatment (Figure 7(a)). Parasite survival after reduction of the *Sm16* transcript levels was evaluated *in vitro* for 10 days. When parasites treated with *Sm16* dsRNA were compared to untreated control or to parasites treated with *GFP* dsRNA, no increment in the percentage of dead schistosomula was observed (Figure 7(b)). With regard to the impact of *Sm16* knockdown on parasite morphology, exposure to *Sm16* dsRNA resulted in a reduction of schistosomula size at days 2 (4.07%) and 4 (5.11%) after dsRNA treatment in comparison to the untreated group (Figures 7(c) and 7(d)), but by the sixth day postexposure to dsRNA, no significant differences in parasite morphology were observed among groups (Figure 7(e)). In order to evaluate the impact of reduced expression of the *Sm16* transcript on the establishment of infection *in vivo*, mice were subcutaneously infected with untreated schistosomula or with schistosomula treated with specific *Sm16* dsRNA or nonspecific *GFP* dsRNA. Fifty days after schistosomula inoculation, no differences in parasite burden or in the number of eggs trapped in the liver or intestine were observed (Table 3).

## 4. Discussion

In order to survive in the definitive host, schistosomes have evolved several immune evasion mechanisms [5]. In the skin, where the parasite first faces the host immune system, an inflammatory response is rapidly regulated by molecules secreted by the parasite [7]. Among these molecules, Sm16, also named SPO-1 or SmSLP, has been described as the major immune modulatory molecule secreted by the parasite during its penetration into the host skin [10]. Due to its potential role in parasite establishment in the host, Sm16 represents a potential target for immunization-based therapies that are aimed at eliminating the parasite.

Therefore, in this study, we produced a recombinant form of Sm16 to be used as an antigen in vaccine formulations against schistosomiasis. Sm16 has been described in the literature as a difficult protein to express in heterologous systems [15, 16, 20]. The first 22 amino acids in the N-terminal of the protein correspond to a signal peptide that drives the protein to a secretory pathway [20]. The C-terminal peptide sequence from Lys^91^ to Gly^94^ of Sm16 has also been described to promote protein aggregation, and a high yield of recombinant protein could only be obtained when the coding region for the amino acids 23-90 of Sm16 was used [15]. So, in order to obtain high yield of rSm16, we choose to express the protein region containing amino acids 23-90. The predicted molecular weight of this Sm16 recombinant protein was 11.5 kDa, but the recombinant protein showed a slow migration pattern in SDS-PAGE, as already observed for other recombinant Sm16 described by other groups [16]. Western blotting analysis of protein extracts from different parasite life-cycle stages demonstrated that Sm16 is not expressed in the adult worm or eggs, but it is more expressed in cercariae and recently transformed schistosomula, consistent with its potential biological function of modulating host immune response during skin migration [10]. Interestingly, Sm16 expression was also detected in 7-day schistosomula. Our results on Sm16 expression in parasite life-cycle stages in the vertebrate host differed from those published by Rao and Ramaswamy [16], which demonstrated Sm16 expression in adult worms. Unlike Rao and Ramaswamy [16], who raised their polyclonal anti-Sm16 antibody using purified native Sm16 as the antigen, in our study, we used polyclonal antibodies produced against recombinant Sm16, which guarantees the specific recognition of this protein in parasite extract.

Staining of whole fixed parasites with polyclonal antibodies against rSm16 demonstrated that a considerable amount of this protein is located on the parasite surface. Sm16 is secreted by the acetabular gland during skin penetration [11] and is described as being able to interact with plasma membrane lipids, in a non-cell-specific manner [20]. This ability to interact with plasma membrane lipids may explain the location of Sm16 at the schistosomula surface, but if this interaction plays any specific role in parasite development or is only a consequence of the ability of Sm16 to interact with lipids still needs further investigation. However, it is worth noting that schistosomula which had Sm16 expression knocked down by RNAi showed reduced parasite size after two and four days in culture, suggesting that Sm16 may interact with a signaling pathway involved in parasite morphology and growth.

Sm16 is the major component of the excretory/secretory products involved in modulating the host immune response in order to promote parasite survival in the skin [10]. Therefore, blocking the biological function of Sm16 could result in protection of the vertebrate host against parasite infection, and antibodies against this antigen could be important factors that block the interaction of this protein with its target in the host or directly neutralize its function. On the other hand, as Sm16 was observed at the parasite surface, an ADCC mechanism could also promote parasite death. This mechanism of death has been previously described to be involved in the protective immunity induced by Smp-80 and GST immunization [28–30]. Thus, to test the ability of vaccine formulations containing rSm16 to induce protection in mice, two different adjuvants were evaluated: Freund's and alum. Freund's adjuvant has been described to induce a Th1 type of immune response, while alum induces a Th2 immune profile [31]. Indeed, we found that mouse immunization with these adjuvants in the absence of antigen induced different immunological profiles. Freund's adjuvant induced significantly higher levels of circulating cytokines (IL-17, TNF-*α*, IFN- ɣ, IL-6, and IL-4) than alum. The addition of the rSm16 to Freund's formulation induced a significant increase in the concentration of circulating IL-2 cytokine, whereas the Sm16/alum formulation did not induce change in the levels of any circulating cytokines evaluated. The cellular profile induced in mice by immunization was also different between the two vaccine formulations. The proportion of circulating B cells induced by the Sm16/alum formulation was higher than that by the Sm16/Freund's formulation. Nevertheless, the opposite was observed with regard to the proportion of memory B cells. The proportion of circulating CD4^+^ T cells and CD4^+^ effector T cells was also lower using the Sm16/alum formulation in comparison with the Sm16/Freund formulation.

Regarding specific antibody production, both vaccine formulations induced significant production of IgG, IgG1, and IgG2a anti-Sm16 antibodies, while the Sm16/alum formulation also induced the production of at least two-fold higher titers of specific IgG and IgG1 antibodies than the Sm16/Freund's formulation. Overall, these results demonstrate that the use of adjuvants in association with rSm16 was able to circumvent the immunosuppressive properties previously demonstrated for the native parasite protein, activating both cellular and humoral immune responses in vaccinated animals.

Neither the immune response triggered by Sm16/alum nor the one triggered by Sm16/Freund formulation was able to induce significant levels of protection in mice. This lack of protection cannot be attributed to the production of nonfunctional antibodies against the native form of the protein, since antibodies produced after immunization recognized the native form of the protein in protein extracts from different parasite stages, and on the schistosomula surface. But, as the C-terminal of Sm16 is lacking in our recombinant protein and since this terminal portion had been described to interact with host immune cells [15, 20], the lack of antibodies against the epitopes from this region of the protein could explain the lack of protection observed in our study. However, this hypothesis still needs to be investigated.

Analysis of the granuloma area in the liver of immunized mice demonstrated that larger granuloma sizes could be observed in mice immunized with Sm16/Freund's. Since Sm16 was not detected in the egg protein extract, this increase in the granuloma area could not be directly attributed to a response against this antigen, but rather to the immune profile observed in animals that received this formulation. Many cytokines have been described to be involved in granuloma formation and modulation. Type-2 cytokines, such as IL-5, IL-4, and IL-13, are responsible for the recruitment and activation of immune cells involved in granuloma formation, whereas IL-10 has been related to the granuloma modulation [32–34]. Another important cytokine involved in the genesis of granuloma is IL-2. Studies have shown that the blockage of this cytokine by neutralizing antibodies results in a reduction of the granuloma area associated with a decrease in IL-5 secretion by T cells [35]. The significantly greater amounts of IL-2 detected in the blood of mice immunized with Sm16/Freund's may be associated with the increased granuloma area observed in animals receiving this vaccine formulation.

Since the immune response triggered by Sm16 immunization did not successfully reduce *S. mansoni* infection, we choose to evaluate the role of this protein in parasite survival during the course of infection. The use of RNAi technology significantly reduced the levels of Sm16 transcripts in schistosomula in comparison to the untreated group from day 1 until day 7 post-dsRNA exposure. Despite having analyzed the levels of RNA, this result is in agreement with the observation that this protein is expressed even in 7-day schistosomula, as observed in the Western blotting and immunofluorescence assays, suggesting that Sm16 expression still occurs in lung-stage parasites. Sm16 knockdown resulted in a reduction of schistosomula size *in vitro* but had no effect on *in vivo* parasite survival or egg production. Besides Sm16, other molecules secreted by the parasite during skin migration have also been described as potential immune modulators of the host immune response [12, 36], including prostaglandin E_2_ (PGE2), SmGST-28 kDa, cyclophilin, and paramyosin [9, 37–40]. In the absence of the Sm16 expression, these proteins may modulate the host immune system to guarantee successful migration of schistosomula through the host skin.

In conclusion, although Sm16 could be detected on the parasite surface by antibodies produced in response to immunization using rSm16, neither the humoral nor the cellular immune responses induced by the rSm16 antigen promoted protection against infection in mice. However, since the recombinant protein used in the vaccine formulations tested in this study did not express the C-terminal part of Sm16, additional studies evaluating the Sm16 C-terminal part are still required before ruling out Sm16 as a vaccine candidate. Our study also demonstrated that decreased Sm16 gene expression in schistosomula had no significant impact in parasite survival and egg laying, suggesting that this protein is not essential for parasite survival or reproduction.

Overall, our results suggest that due to the redundancy of parasite immunosuppressive molecules in the evasion process, a vaccine that seeks to neutralize the effect of these molecules should include several vaccine targets in its formulation.

## Figures and Tables

**Table 1:** Endpoint titers of specific IgG antibodies against rSm16 in immunized mice.

	1^st^ dose			2^nd^ dose			3^rd^ dose		
	IgG	IgG1	IgG2a	IgG	IgG1	IgG2a	IgG	IgG1	IgG2a
rSm16/Freund's	6,400	1,600	200	51,200	25,600	1,600	204,800	102,400	3,200
rSm16/alum	25,600	12,800	200	102,400	102,400	3,200	408,600	204,800	3,200

**Table 2:** Protection induced by rSm16 immunization in mice.

	Worm burden			Protection (%)	Eggs/gram of liver (mean ± SD)	Egg/gram of intestine (mean ± SD)
	Male	Female	Total			
Trial 1						
Freund's	24 ± 6	21 ± 3.4	45 ± 8.1		10,604 ± 4,197	nd
rSm16/Freund's	23 ± 4	22 ± 5.5	46 ± 9.8	0%	20,151 ± 9,698	nd
Alum	25 ± 7.6	23 ± 7.5	43 ± 13		27,796 ± 18,446	22,176 ± 8,138
rSm16/alum	20 ± 6.5	18 ± 5.7	40 ± 10	7.0%	14,870 ± 10,373	16,353 ± 4,229
Trial 2						
Freund's	24 ± 8.6	22 ± 8	46 ± 18		40,088 ± 13,835	25,451 ± 11,114
rSm16/Freund's	26 ± 6.4	21 ± 6	47 ± 14	0%	56,091 ± 21,221	39,673 ± 23,297
Alum	21 ± 6.8	18 ± 6	39 ± 11		22,019 ± 6,143	16,009 + 6,931
rSm16/alum	25 ± 6.5	21 ± 6.1	46 ± 12	0%	23,806 ± 9,331	14,111 + 2,009

nd: not determined.

**Table 3:** Reduction in the parasite burden from mice infected with *Sm16*-silenced schistosomula.

	Worm burden recovery (mean ± SD)	% reduction^∗^	Eggs/gram of liver (mean ± SD)	% reduction^#^	Eggs/gram of intestine (mean ± SD)	% reduction^#^
Trial 1						
Control	25 ± 17		10,336 ± 4,813		3,673 ± 1,569	
*GFP*	30 +/-14	NS	10,274 ± 5,153	NS	6,928 ± 4,290	NS
*Sm16*	23 ± 13	8% (NS)	9,445 ± 4,413	8.6% (NS)	4,595 ± 2,934	NS
Trial 2						
Control	19 ± 17		10,435 ± 9,528		3,156 ± 3,575	
*GFP*	18 ± 10	5.3% (NS)	10,388 ± 6,407	0.5% (NS)	2,195 ± 1,460	30% (NS)
*Sm16*	25 ± 20	NS	12,941 ± 6,536	NS	4,679 ± 4,305	NS

^∗^Reduction of total worms compared to control group. ^#^Reduction of eggs in tissue (liver and intestine) compared to the control group. NS: not significant.

## Data Availability

The data used to support the findings of this study are included within the article.

## Supplementary Materials

Supplementary Materials Supplementary Figure 1: immunization protocol. Supplementary Figure 2: Sm16-specific antibody titer endpoints in immunized mouse serum. Pools of sera from mice immunized with rSm16 plus Freund's adjuvant obtained 15 days after the second dose (black circles) or 30 days after the third dose (black square) were serially diluted, beginning at 1 : 50, and used in an ELISA assay. The threshold was calculated using the mean absorbance value of the blank wells plus two standard deviations. The arrows indicate antibody titer endpoints observed after two and three immunization doses.
