# Supplementary material for: Sm16, A Schistosoma mansoni Immunomodulatory Protein, Fails to Elicit a Protective Immune Response and Does Not Have an Essential Role in Parasite Survival in the Definitive Host
Source: J Immunol Res. 2019 Dec 1;2019:6793596. doi: 10.1155/2019/6793596 (PMC6915009; doi:10.1155/2019/6793596)
Supplement: Supplementary Materials — Supplementary Figure 1: immunization protocol. Supplementary Figure 2: Sm16-specific antibody titer endpoints in immunized mouse serum. Pools of sera from mice immunized with rSm16 plus Freund's adjuvant obtained 15 days after the second dose (black circles) or 30 days after the third dose (black square) were serially diluted, beginning at 1 : 50, and used in an ELISA assay. The threshold was calculated using the mean absorbance value of the blank wells plus two standard deviations. The arrows indicate antibody titer endpoints observed after two and three immunization doses. [file 6793596.f1.pdf]

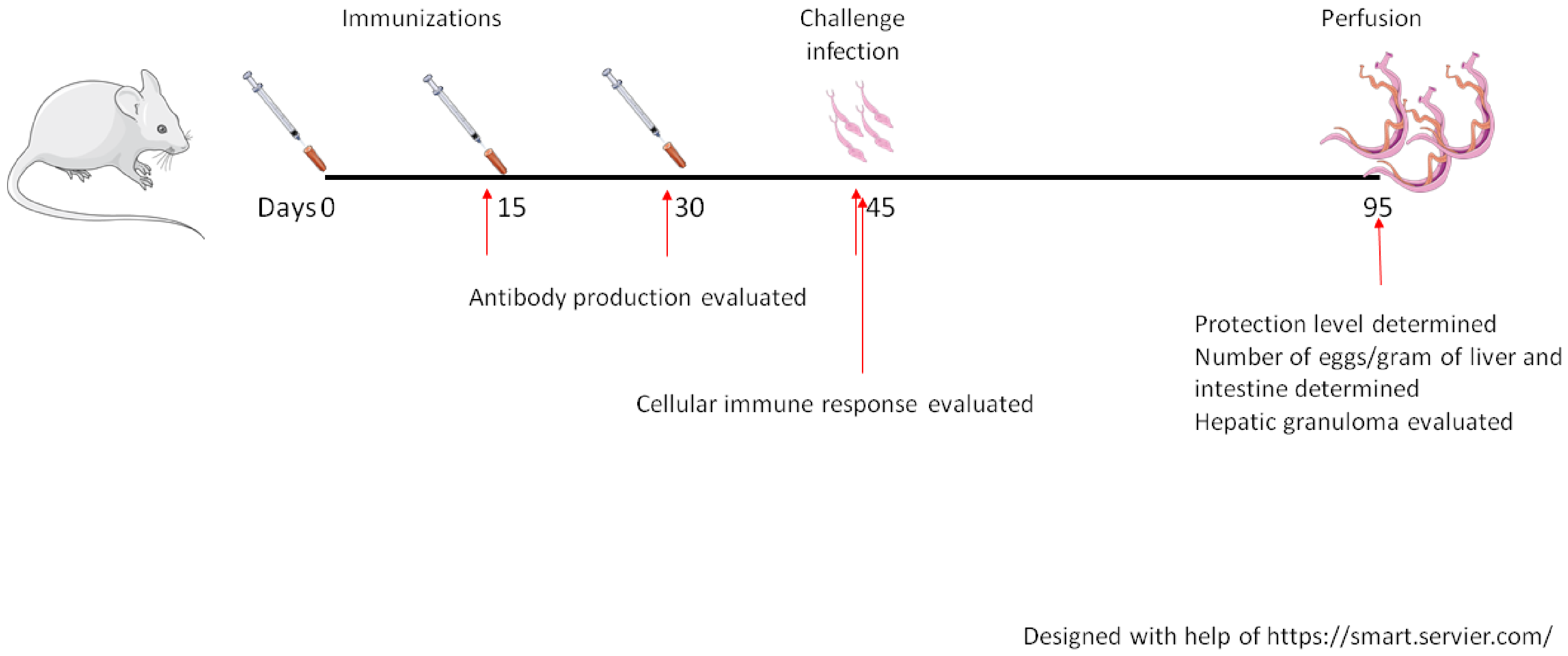

Supplementary Figure 1: Immunization protocol.

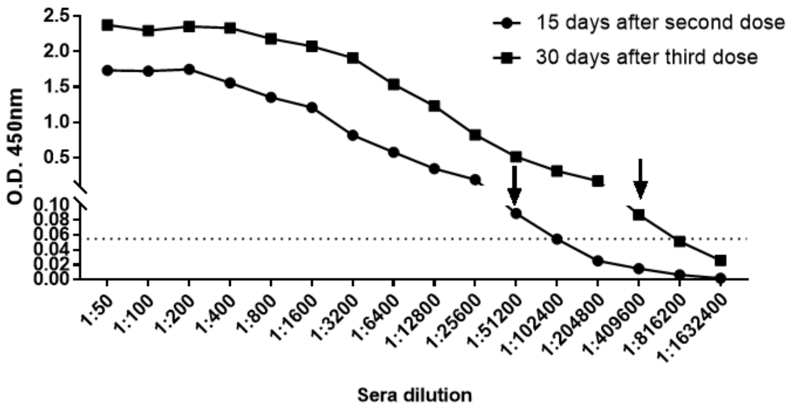

Supplementary Figure 2. **Sm16-specific antibodies titer endpoints in immunized mice serum.** Pools of sera from mice immunized with rSm16 plus Freund's adjuvant obtained 15 days after the second dose (black circles) or 30 days after the third dose (black square) were serially diluted, beginning at 1:50, and used in an ELISA assay. The threshold was calculated using the mean absorbance value of the blank wells plus two standard deviations. The arrows indicate antibodies titer endpoints observed after two and three immunization doses.
